# Synaptotagmin-1 and -7 Are Redundantly Essential for Maintaining the Capacity of the Readily-Releasable Pool of Synaptic Vesicles

**DOI:** 10.1371/journal.pbio.1002267

**Published:** 2015-10-05

**Authors:** Taulant Bacaj, Dick Wu, Jacqueline Burré, Robert C. Malenka, Xinran Liu, Thomas C. Südhof

**Affiliations:** 1 Department of Molecular and Cellular Physiology and Howard Hughes Medical Institute, Stanford University, Stanford, California, United States of America; 2 Nancy Pritzker Laboratory, Department of Psychiatry and Behavioral Sciences, Stanford University Medical School, Stanford, California, United States of America; 3 Department of Cell Biology, Yale University School of Medicine, New Haven, Connecticut, United States of America; Princeton University, UNITED STATES

## Abstract

In forebrain neurons, Ca^2+^ triggers exocytosis of readily releasable vesicles by binding to synaptotagmin-1 and -7, thereby inducing fast and slow vesicle exocytosis, respectively. Loss-of-function of synaptotagmin-1 or -7 selectively impairs the fast and slow phase of release, respectively, but does not change the size of the readily-releasable pool (RRP) of vesicles as measured by stimulation of release with hypertonic sucrose, or alter the rate of vesicle priming into the RRP. Here we show, however, that simultaneous loss-of-function of both synaptotagmin-1 and -7 dramatically decreased the capacity of the RRP, again without altering the rate of vesicle priming into the RRP. Either synaptotagmin-1 or -7 was sufficient to rescue the RRP size in neurons lacking both synaptotagmin-1 and -7. Although maintenance of RRP size was Ca^2+^-independent, mutations in Ca^2+^-binding sequences of synaptotagmin-1 or synaptotagmin-7—which are contained in flexible top-loop sequences of their C2 domains—blocked the ability of these synaptotagmins to maintain the RRP size. Both synaptotagmins bound to SNARE complexes; SNARE complex binding was reduced by the top-loop mutations that impaired RRP maintenance. Thus, synaptotagmin-1 and -7 perform redundant functions in maintaining the capacity of the RRP in addition to nonredundant functions in the Ca^2+^ triggering of different phases of release.

## Abstract 2

Synaptotagmins function not only as calcium sensors in triggering synaptic vesicle fusion but also contribute to the preparation of synaptic vesicles for fusion, being required for full priming of vesicles ready for subsequent triggering of exocytosis.

## Author Summary

Neurons communicate with each other at specialized contact points called synapses. Presynaptic neurons store chemical neurotransmitters within presynaptic vesicles at the nerve terminal. During synaptic transmission, the presynaptic vesicles fuse with the plasma membrane, releasing their neurotransmitter content into the synaptic cleft to activate postsynaptic receptors. Neurotransmitter release is a multistage process that requires the priming of synaptic vesicles into a readily-releasable pool of vesicles. When an action potential—a transient electrical signal that travels along the neuron—invades a nerve terminal, it promotes the influx of extracellular calcium ions (Ca^2+^) that, in turn, trigger fusion of primed vesicles, thereby causing neurotransmitter release. Previous studies established that synaptotagmins function as Ca^2+^ sensors for release and, additionally, inhibit spontaneous fusion of synaptic vesicles in the absence of an action potential. In most neurons of the anterior part of the brain, two synaptotagmins, synaptotagmin-1 and -7, mediate fast and slow neurotransmitter release, respectively. We now show that in addition to their nonoverlapping roles as Ca^2+^ sensors and fusion clamps, synaptotagmin-1 and -7 perform an essential overlapping function in maintaining the readily-releasable pool of vesicles. This function is redundantly performed by both synaptotagmins; therefore, an impairment of the readily-releasable pool manifests only when both synaptotagmins are deleted. These results extend the functions of synaptotagmins to steps upstream of Ca^2+^ triggering of release and suggest that synaptotagmins, despite their simple domain structure, perform multiple sequential roles in neurotransmitter release. Thus, synaptotagmins coordinate multiple stages of Ca^2+^-triggered exocytosis, ensuring fast synaptic transmission for rapid information transfer between neurons at synapses.

## Introduction

Synaptic vesicles are released within a few hundred microseconds of Ca^2+^ influx into a presynaptic terminal [1,2]. Exocytosis of synaptic vesicles is carried out by neuronal soluble NSF-attachment protein receptor (SNARE) and Sec1/Munc18-like (SM) proteins and triggered by Ca^2+^ binding to synaptotagmins [3]. To prepare for rapid exocytosis with millisecond temporal precision, synaptic vesicles undergo a series of maturation steps that result in the formation of the readily-releasable pool (RRP) of vesicles poised for Ca^2+^-triggered exocytosis.

The first step that prepares synaptic vesicles for rapid exocytosis is the recruitment of vesicles to the active zone (“tethering”). Morphologically, tethered vesicles abut the plasma membrane when examined by standard electron microscopy (EM) of chemically fixed tissues [4]. After tethering, vesicles undergo a priming process that firmly docks the vesicles at the active zone, as confirmed by EM of unfixed samples subjected to high-pressure freezing, which suggested that priming directly attaches vesicles to the presynaptic active zone downstream of tethering [4,5]. As a result, mutations that impair priming cause a loss of vesicle docking when viewed in rapidly frozen unfixed samples, whereas these mutations appear to have no effect on vesicle tethering when chemically fixed samples are examined [4–7]. Strikingly, the only known mutation in mammalian synapses that alters vesicle tethering as viewed in chemically fixed samples is the deletion of Rab3-interacting molecules (RIMs), which are active zone proteins that mediate vesicle tethering by binding to Rab3 and Rab27 proteins on synaptic vesicles [8–11].

Priming of synaptic vesicles produces the RRP of vesicles whose capacity is generally measured by monitoring neurotransmitter release induced by hypertonic sucrose, which stimulates synaptic vesicle fusion by a Ca^2+^-independent, mechanical mechanism [12]. Two classes of priming factors were identified: active zone proteins such as Munc13 and RIM that are involved in organizing the machinery for synaptic vesicle exocytosis [7,13,14], and SNARE and SM proteins that mediate membrane fusion of synaptic vesicles during exocytosis [15–21]. A widely accepted model suggests that priming results in the partial assembly of SNARE and SM protein complexes, an assembly that is catalyzed by active zone priming factors, but the mechanisms that determine the capacity of the RRP are poorly understood [3].

Extensive studies demonstrated that three vertebrate synaptotagmins (synaptotagmin-1 [Syt1], Syt2, and Syt9) act as Ca^2+^ sensors for fast neurotransmitter release [22–25]. Syt1, Syt2, and Syt9 are generally expressed in different populations of neurons, although some overlap in expression exists, with most rostral brain neurons expressing only Syt1 and most caudal brain neurons expressing Syt2 [26]. Recent results revealed that a slower and less prominent form of Ca^2+^-triggered release, which becomes dominant in synapses lacking Syt1, is mediated at least in part by synaptotagmin-7 (Syt7), which is abundantly coexpressed with Syt1, Syt2, and Syt9 in nearly all neurons [27–29]. A role of Syt7 as a Ca^2+^ sensor for slow synaptic vesicle exocytosis is supported by findings in neuroendocrine cells where Syt7, like Syt1, localizes to secretory granules and mediates Ca^2+^-induced exocytosis with slower kinetics than Syt1 [30–38].

Viewed together, these observations seem to suggest a linear progression of synaptic vesicle exocytosis from tethering to priming to Ca^2+^ triggering of fusion, with synaptotagmins mediating the Ca^2+^-triggering step. Consistent with this hypothesis, individual deletions of Syt1, Syt2, or Syt7 had no effect on the size of the RRP [22,24,28,39]. However, three puzzling observations were difficult to reconcile with this hypothesis and suggested synaptotagmin also functions upstream of Ca^2+^ triggering. First, loss-of-function of complexin, which is an essential cofactor of all synaptotagmins in Ca^2+^ triggering of fusion [40–44], decreases the size of the RRP approximately 2–3-fold [45–47]. Second, blocking Syt1 or Syt2 function (but not Syt7 function) caused an increase in spontaneous “mini” release in a manner that suggested a role of Syt1 and Syt2 in clamping minis upstream of Ca^2+^ triggering [24,26,39,48–50]. Ablation of complexin also increased spontaneous mini release at least in some preparations, suggesting a common mode of action of Syt1 or Syt2 with complexin in clamping mini release [3]. Third, immunoprecipitations and pulldowns indicated that Syt1 interacts with SNARE proteins not only in a Ca^2+^-dependent manner but also in a Ca^2+^-independent manner [51–54]. Viewed together, these findings raised the possibility that synaptotagmins may perform additional functions besides Ca^2+^ triggering of release despite the lack of an effect of individual synaptotagmin deletions on RRP size.

Based on these observations, we reasoned that synaptotagmins may have a role in exocytosis upstream of Ca^2+^ triggering by maintaining the RRP, and that this role may have been overlooked in analyses of single Syt1, Syt2, and Syt7 knockout (KO) neurons because Syt1 and Syt2 might be functionally redundant with Syt7 for priming of exocytosis, even though they are not functionally redundant with Syt7 for Ca^2+^ triggering. We thus tested in forebrain neurons (which express little Syt2 or Syt9) whether simultaneous ablation of both Syt1 and Syt7 impacts the RRP. We found that loss-of-function of both Syt1 and Syt7 indeed decreased the RRP size ~2–3-fold. Moreover, we observed that the redundant function of Syt1 and Syt7 in maintaining the RRP size requires the flexible top-loop sequences of the Syt1 or Syt7 C2 domains that form their Ca^2+^ binding sites, even though we confirmed that maintenance of the normal RRP size itself does not require Ca^2+^. We show that the Syt1 and Syt7 double loss-of-function does not detectably alter the rate of vesicle priming into the RRP, despite the fact that it dramatically decreases the RRP size. Furthermore, we demonstrate that Syt1 and Syt7 bind to SNARE complexes, that Syt1 increases SNARE complex assembly in the presence of complexin, and that Syt1- and Syt7-binding to SNARE complexes is impaired by the top-loop Ca^2+^-binding sequence mutations. Our data suggest that at a given synapse, different synaptotagmins mediate distinct phases of Ca^2+^ triggering of neurotransmitter release but redundantly maintain the capacity of the RRP upstream of Ca^2+^ triggering, thus enabling the organization of a fast and efficient release machinery at the synapse.

## Results

### Simultaneous Loss-of-Function of Syt1 and Syt7 Impairs the RRP at Inhibitory and Excitatory Synapses

**Fig 1:**
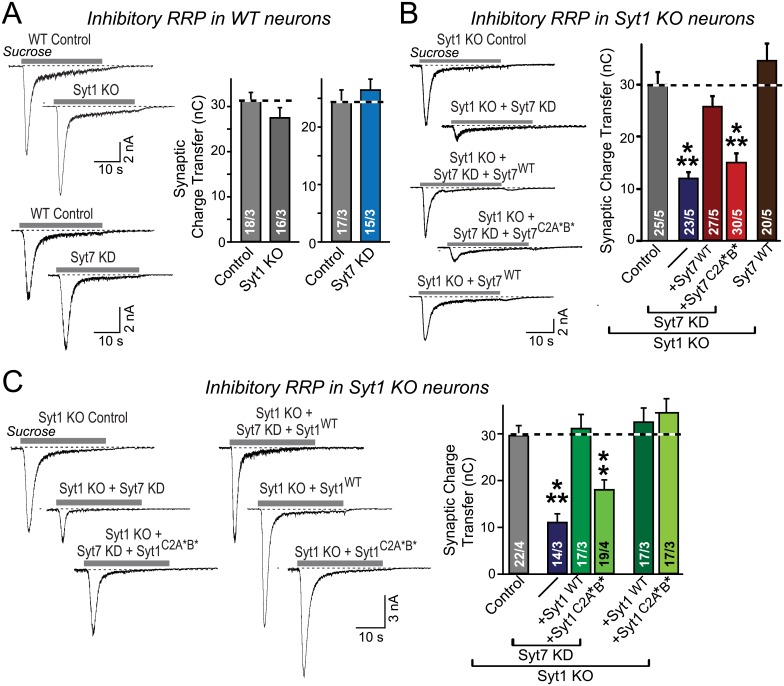
Simultaneous ablation of Syt1 and Syt7 decreases the RRP size at inhibitory synapses: Rescue by WT but not mutant Syt1 or Syt7. **A.** Ablation of either Syt1 or Syt7 alone does not alter the RRP at inhibitory synapses. Hippocampal neurons were cultured from littermate WT and Syt1 KO mice (for Syt1 analysis) or from WT mice and were then infected at DIV4 with control or Syt7 KD lentiviruses (for Syt7 analysis). At DIV14–16, exocytosis of primed vesicles from the RRP in the neurons was stimulated by application of 0.5 M sucrose for 30 s (gray bars in representative traces on the left), and the RRP size was estimated as the synaptic charge transfer integrated over 30 s (summary graphs on the right). Recordings were performed in the presence of 1 μM tetrodotoxin, 20 μM CNQX, and 50 μM AP5 to isolate inhibitory currents. **B.** Simultaneous ablation of Syt1 and Syt7 decreases the RRP size of inhibitory synapses in a manner that is rescued by WT Syt7 (Syt1^WT^) but not Syt7 with mutations in the top C2 domain sequences containing the Syt7 Ca^2+^ binding sites (Syt7^C2A^*^B^*). Hippocampal Syt1 KO neurons were infected with control lentiviruses, Syt7 KD lentiviruses without or with expression of either WT or mutant Syt7, or lentiviruses only expressing WT Syt7 (as a control for overexpression effects). RRP was measured as described in A. **C.** The decreased RRP size in Syt1/7 double-deficient neurons is rescued by WT Syt1 (Syt1^WT^) but not Syt1-containing mutations in the top-loop Ca^2+^-binding sequences (Syt1^C2A^*^B^*). Experiments were performed as described for B. All data are means ± SEM (Standard Error of the Mean); numbers in bars indicate number of neurons/independent cultures analyzed. Statistical significance was assessed by one-way ANOVA (** *p* < 0.01; *** *p* < 0.001). The data used to make this figure can be found in S1 Data.

We measured the presynaptic RRP size in cultured hippocampal neurons as the synaptic charge transfer induced by a brief (10–30 s) application of hypertonic sucrose [12]. Consistent with earlier studies [22,28,39], individual Syt1 or Syt7 KO or knockdown (KD) manipulations had no effect on RRP size (Fig 1A). When we tested hippocampal neurons that lacked both Syt1 and Syt7, however, we observed a ~60% decrease in inhibitory synaptic transmission, quantified both as the synaptic charge transfer during the initial transient of the inhibitory postsynaptic current (IPSC) and as the synaptic charge transfer over the entire period of sucrose application (Fig 1B and 1C). The decrease in RRP size was reversed by reintroduction of wild-type (WT) Syt1 or Syt7 but not of mutant Syt1 or mutant Syt7 containing amino acid substitutions in the top-loop sequences of both C2 domains, which include their Ca^2+^ binding sites (Syt1^C2A^*^B^* and Syt7^C2A^*^B^*; Fig 1B and 1C). Overexpression of Syt1 or Syt7 rescue proteins in Syt1 KO neurons, tested as a control, had no effect on RRP size (Fig 1B and 1C). The lack of rescue by the mutant Syt1 and Syt7 proteins was not due to impaired mutant protein expression, because we previously showed that these mutant proteins are well expressed in neurons [28].

**Fig 2:**
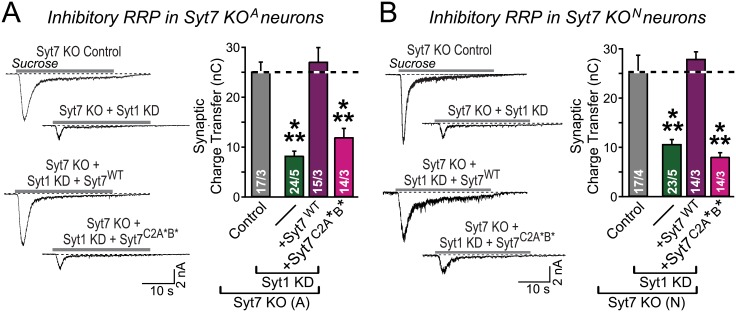
KD of Syt1 in Syt7 KO neurons decreases the RRP size at inhibitory synapses: Rescue by WT but not mutant Syt7. **A & B.** Same as 1B, except that Syt7 KO neurons with a Syt1 KD were examined. Hippocampal neurons were cultured from two independent, constitutive Syt7 KO mouse lines (D, Syt7 KO^A^ from [56]; E, Syt7 KO^N^ from [55]), and infected with control lentivirus or Syt1 KD lentivirus without or with superinfection with a second lentivirus expressing WT or mutant Syt7. All data are means ± SEM; numbers in bars indicate number of neurons or independent cultures analyzed. Statistical significance was assessed by one-way ANOVA (** *p* < 0.01; *** *p* < 0.001). The data used to make this figure can be found in S1 Data.

To rule out the possibility of off-target effects by the Syt7 shRNA used for the Syt7 KD in these experiments, we performed the reverse experiment. We cultured hippocampal neurons from two different Syt7 KO mouse lines [55,56] and measured the RRP size as a function of a Syt1 KD [28,57]. As expected, Syt7 KO neurons containing Syt1 exhibited a normal RRP size measured as the total sucrose-evoked charge transfer. KD of Syt1 in the Syt7 KO neurons, however, severely depressed the RRP of inhibitory synapses in both Syt7 KO mouse lines (Fig 2A and 2B). Similar to the Syt1 KO findings, re-expression of WT Syt7, but not of mutant Syt7^C2A^*^B^*, restored the RRP size in Syt7 KO/Syt1 KD neurons (Fig 2A and 2B). Taken together, these experiments rule out off-target effects as the origin of the observed RRP phenotype in Syt1/7 double-deficient neurons.

**Fig 3:**
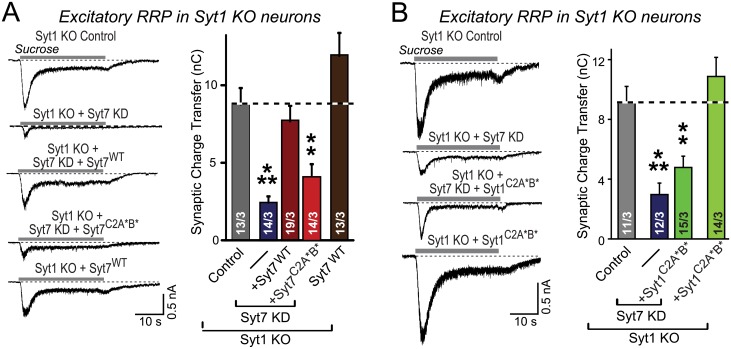
Simultaneous ablation of Syt1 and Syt7 decreases the RRP size at excitatory synapses. **A & B.** Ablation of both Syt1 and Syt7 in hippocampal neurons decreases the RRP size at excitatory synapses in a manner that is rescued by WT Syt7 (Syt7^WT^) or Syt1 (Syt1^WT^) but not by mutant Syt7 (Syt7^C2A^*^B^*) or Syt1 (Syt1^C2A^*^B^*) with altered top-loop sequences containing the Ca^2+^-binding sequences (A, Syt7 rescue; B, Syt1 rescue). Experiments were performed as described for Fig 1, except that picrotoxin (50 μM) was used instead of CNQX. All data are means ± SEM; numbers in bars indicate number of neurons/independent cultures analyzed. Statistical significance was assessed by one-way ANOVA (** *p* < 0.01, *** *p* < 0.001). The data used to make this figure can be found in S1 Data.

To assess whether the redundant requirement for Syt1 and Syt7 in maintaining the RRP size is a general feature of synapses, we next measured the RRP at excitatory synapses as the excitatory postsynaptic current (EPSC) evoked by hypertonic sucrose. We found that the RRP size in excitatory synapses was reduced to the same extent by the Syt1 KO/Syt7 KD as in inhibitory synapses (~60%), and that this reduction could also be rescued by re-introduction of WT Syt7 but not of mutant Syt1 or Syt7 (Fig 3A and 3B). Thus, Syt1 and Syt7 are redundantly required for RRP maintenance in both excitatory and inhibitory forebrain synapses.

The observation that mutations in the top-loop sequences of Syt1 and Syt7 (which also contain their Ca^2+^ binding sites) blocked their ability to sustain vesicle priming (Figs 1–3) was surprising because priming of vesicles into the RRP is thought to proceed by a Ca^2+^-independent mechanism [12]. To confirm that priming as measured by sucrose-induced synaptic transmission under our conditions is indeed Ca^2+^-independent, we incubated cultured neurons for 30 min in a bath solution containing 10 mM BAPTA-AM. This manipulation blocks nearly all spontaneous miniature synaptic events (i.e., miniature EPSCs [mEPSCs] and miniature IPSCs [mIPSCs]), which are mostly Ca^2+^-dependent [49]. When we then assessed the RRP, we found that the BAPTA-AM did not decrease the RRP (S1 Fig). Thus, RRP maintenance in our conditions does not require Ca^2+^, not even at resting levels.

### Syt1/Syt7 Double Deficient Neurons Exhibit No Major Changes in Synaptic Ultrastructure

**Fig 4:**
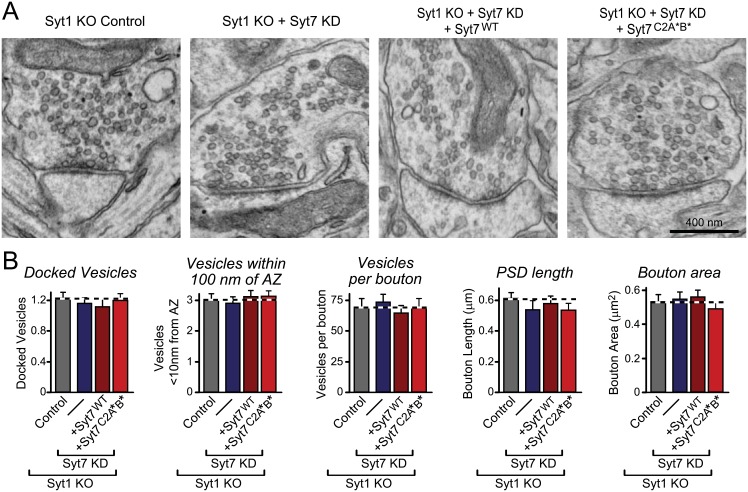
Syt1/Syt7 double-deficiency does not induce structural changes in synapses as assessed by standard EM. **A.** Representative electron micrographs from cultured hippocampal Syt1 KO neurons that were infected with control lentivirus or Syt7 KD lentiviruses without or with expression of WT or mutant Syt7 rescue cDNAs. **B.** Quantification of the total number of docked vesicles (defined as vesicles touching the membrane), vesicles at the active zone (AZ, defined as vesicles within 100 nm of plasma membrane), total number of vesicles in each bouton, and postsynaptic density (PSD) length and bouton area in EM micrographs. Quantification from two independent cultures was performed manually by an observer blind to the conditions. No significant differences were found for the parameters analyzed. The data used to make this figure can be found in S1 Data.

The phenotype of an apparently decreased RRP size in Syt1/7 double-deficient neurons, as revealed by the results up to this point, could be caused by an impairment in vesicle priming, a decrease in the capacity of the RRP, a decrease in the number of synapses because the Syt1/7 deficiency may produce a synapse loss, and/or a change in the ultrastructural organization of synapses. We previously showed that synapse numbers are unaltered in Syt1/7 double-deficient neurons, ruling out a loss of synapses as a cause of the phenotype [28]. To address the possibility that the simultaneous ablation of Syt1 and Syt7 alters the architecture of the nerve terminal, we performed transmission EM of cultured neurons (Fig 4). We observed no effect of the Syt1/Syt7 double loss-of-function on any basic ultrastructural parameter of synapses examined, excluding a major effect on vesicle organization in the terminal. Our results suggest that although Syt1 and Syt7 mediate separate essential functions in Ca^2+^ triggering and are not required for vesicle priming on their own [22,28,29], they perform overlapping functions in maintaining the normal capacity of the RRP of synaptic vesicles. Therefore, Syt1 and Syt7 are nonredundant for Ca^2+^ triggering but redundant for RRP maintenance.

### Syt1 and Syt7 Utilize Distinct C2 Domains for Catalyzing Exocytosis versus Supporting RRP Size

In Syt1, Ca^2+^ triggering of release is mediated primarily by Ca^2+^ binding to the C2B domain, although Ca^2+^ binding to the C2A domain contributes significantly [23,50,58–62]. In Syt7, conversely, Ca^2+^ triggering of release is mediated primarily by the C2A domain [28]. Our current finding that Syt1 and Syt7 perform redundant functions in vesicle priming in addition to nonredundant functions in Ca^2+^ triggering prompted us to ask whether the RRP function of Syt1 and Syt7 involves the same C2 domains with a similarly asymmetric C2 domain requirement.

**Fig 5:**
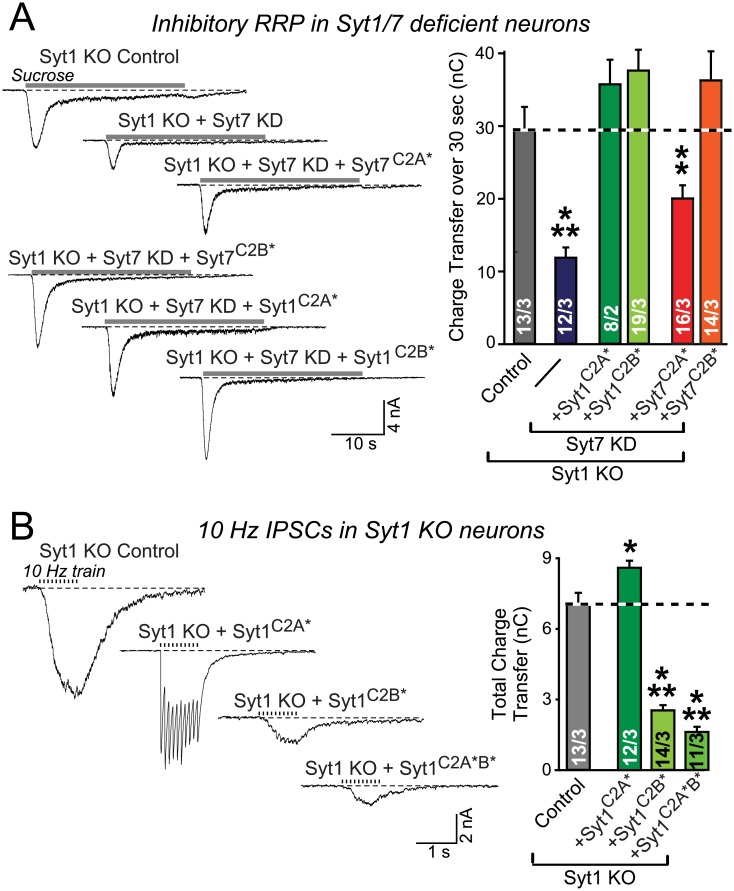
Differential requirement for Syt1 and Syt7 C2A- and C2B-domain top-loop sequences for RRP maintenance and Ca^2+^ triggering of neurotransmitter release. **A.** In Syt1/7 double-deficient neurons, Syt1 mutations in either the C2A-domain (Syt1^C2A^*) or C2B domain (Syt1^C2B^*) top-loop sequences that include their Ca^2+^ binding sites do not block rescue of the RRP in Syt1/7 double deficient neurons, whereas Syt7 mutations in the C2A-domain (Syt1^C2A^*) but not the C2B domain (Syt1^C2B^*) Ca^2+^-binding sequence selectively impair rescue of the RRP. Hippocampal neurons were cultured from Syt1 KO mice and infected with control lentivirus, or Syt7 KD lentiviruses co-expressing the indicated Syt1 and Syt7 mutants. Exocytosis of primed vesicles from the RRP was induced by a brief pulse of 0.5 M sucrose, and monitored as IPSCs in the presence of TTX (1 μM), CNQX (20 μM), and AP5 (50 μM). Representative traces are shown on the left, and summary graphs on the right. Note that simultaneous mutation of both Syt1 C2-domains blocks its priming function (Fig 1C). **B.** Confirmation that mutations of Syt1 in the top-loop sequences containing the Ca^2+^ binding sites of the C2B domain but not the analogous mutations in the C2A domain block rescue of synchronous release in Syt1 KO neurons. Experiments were performed as described for A, except that IPSCs were elicited by 10 Hz, 1 s stimulus trains (left, representative traces; right, summary graphs of the total synaptic charge transfer). Stimulations are indicated by tick marks. All data are means ± SEM; numbers in bars indicate number of neurons/independent cultures analyzed. Statistical significance was assessed by one-way ANOVA (* *p* < 0.05, ** *p* < 0.01, *** *p* < 0.001). The data used to make this figure can be found in S1 Data.

Mutant Syt1 with top-loop substitutions in the Ca^2+^-binding sequence of the C2A domain (Syt1^C2A^*) rescued priming of release in Syt1/Syt7 double-deficient neurons, similar to its ability to partially rescue Ca^2+^-triggered fast release in Syt1 KO neurons (Fig 5A) [58,62]. Surprisingly, however, mutant Syt1 with analogous substitutions in the C2B domain (Syt1^C2B^*) also rescued priming (Fig 5A and 5B). This latter result was unexpected in view of the selectively essential role of the Syt1 C2B domain Ca^2+^-binding sequence in Ca^2+^ triggering of fast release (Fig 5B) [23,58,60,62]. Thus, Syt1 exhibits distinct C2 domain requirements for RRP maintenance and for Ca^2+^ triggering.

In contrast to Syt1, mutant Syt7 with top-loop substitutions in the Ca^2+^-binding sequence of the C2A domain (Syt7^C2A^*) was unable to rescue the RRP phenotype (Fig 5A). The Syt7 C2B domain mutant (Syt7^C2B^*), however, fully rescued priming, although the presence of the C2B domain was required for Syt7 function (Fig 5A and S2 Fig). Therefore in Syt7, the top-loop sequences of the C2A domain but not the C2B domain are selectively required for both priming and triggering of release since we previously showed that the C2A domain mutant also cannot rescue slow Ca^2+^ triggering in Syt1/Syt7-double-deficient neurons [28]. Although the functions of Syt1 and Syt7 in priming are redundant, their mechanisms of action appear to differ in terms of their C2 domain sequences.

We extended these conclusions in further experiments in which we tested point mutations in or next to the Ca^2+^ binding sites of Syt7, and additionally examined swap mutations in which the entire Ca^2+^ binding sequences were exchanged between Syt1 and Syt7 (S3 Fig). We found that the precise sequence of the Syt1 and Syt7 Ca^2+^ binding site was not a critical determinant of their ability to prime vesicles for release or to mediate Ca^2+^ triggering of release, but that a single point mutation adjacent to the Syt7 Ca^2+^ binding sites modestly decreased its priming function. These results corroborate the notion that the top-loop sequences support vesicle priming by a Ca^2+^-independent mechanism.

### Mutations in the Syt1 and Syt7 Top-Loop Sequences That Also Contain Their Ca^2+^-Binding Sites Impair Association with SNARE Complexes

**Fig 6:**
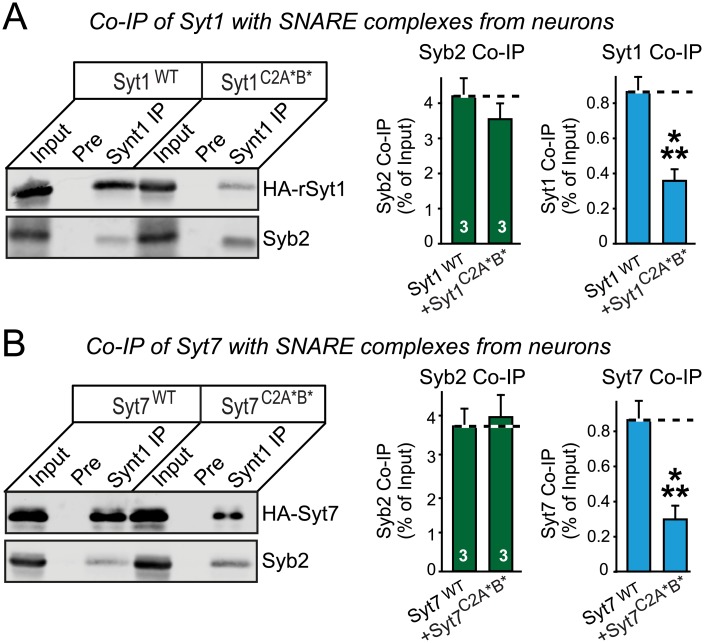
Syt1 and Syt7 both bind to SNARE complexes, and mutations that alter the top-loop Ca^2+^-binding sequences of Syt1 or Syt7 decrease such binding. **A & B.** Analysis of the SNARE binding of WT Syt1 and Syt7 (Syt1^WT^ and Syt7^WT^, respectively) and of mutant Syt1 and Syt7-containing top-loop substitutions altering their Ca^2+^-binding sequences (Syt1^C2A^*^B^* and Syt7^C2A^*^B^*, respectively; A, Syt1^WT^ and Syt1^C2A^*^B^*; B, Syt7^WT^ and Syt7^C2A^*^B^*). WT or mutant Syt1 and Syt7 were lentivirally expressed in neurons, and neurons were analyzed at DIV14–16 by immunoprecipitation of the SNARE protein syntaxin-1 followed by immunoblotting for the SNARE protein synaptobrevin-2 (Syb2; to assess the degree of SNARE complex IP) or of Syt1 or Syt7 (to assess the degree of synaptotagmin binding). Data are means ± SEM (*n* = 3 independent culture experiments). Statistical significance was assessed by one-way ANOVA (*** *p* < 0.001). The data used to make this figure can be found in S1 Data.

How do Syt1 and Syt7 redundantly function in maintaining the RRP size? It is thought that primed vesicles in the RRP are associated with partially or completely assembled SNARE complexes [3], and Syt1 is known to bind to SNARE complexes both in a Ca^2+^-independent and a Ca^2+^-dependent manner [54]. We hypothesized that the requirement for the Syt1 or Syt7 top-loop sequences in priming may be due to an alternative, independent action of these sequences that is unrelated to Ca^2+^ binding, and that may be related to SNARE binding. Two recent studies using nuclear magnetic resonance (NMR) spectroscopy and crystallography to determine how Syt1 binds to SNARE complexes found strong Syt1 binding to SNARE complexes in the absence of Ca^2+^, but detected no major interaction of SNARE complexes with the top-loop Ca^2+^-binding sequences [63,64]. The two studies mapped distinct Syt1 binding sites, however, suggesting that Syt1-binding (and by extension, Syt7-binding) to SNAREs may be multifaceted. To test whether Syt7 also binds to neuronal SNARE complexes and whether the top-loop mutations in Syt1 and/or Syt7 impair such binding, we measured the effect of mutations in the Ca^2+^ binding site sequences in Syt1 and Syt7 on the association of Syt1 and/or Syt7 with SNARE complexes in neurons. We expressed HA-tagged WT or mutant Syt1 or Syt7 in cultured neurons using lentiviruses, and immunoprecipitated the SNARE protein syntaxin-1 from these neurons. We then analyzed the immunoprecipitates by quantitative immunoblotting for the SNARE protein synaptobrevin-2 (to assess SNARE complex assembly) and for Syt1 or Syt7 (to assess binding of Syt1 or Syt7 to syntaxin-1 and/or SNARE complexes; Fig 6A and 6B).

We found that WT Syt1 and Syt7 co-immunoprecipitated with SNAREs to a similar extent, suggesting that both interact with SNARE complexes. The top-loop sequence mutations of Syt1 or Syt7 decreased their coimmunoprecipitations with syntaxin-1 by ~60% but had no effect on the coimmunoprecipitation of synaptobrevin-2 with syntaxin-1 (Fig 6A and 6B). These experiments suggest that Syt1 and Syt7 both bind to SNAREs—either syntaxin-1 alone or SNARE complexes—and that this binding is impaired by the top-loop sequence mutations.

### Syt1 Increases SNARE complex Assembly in the Presence of Complexin

**Fig 7:**
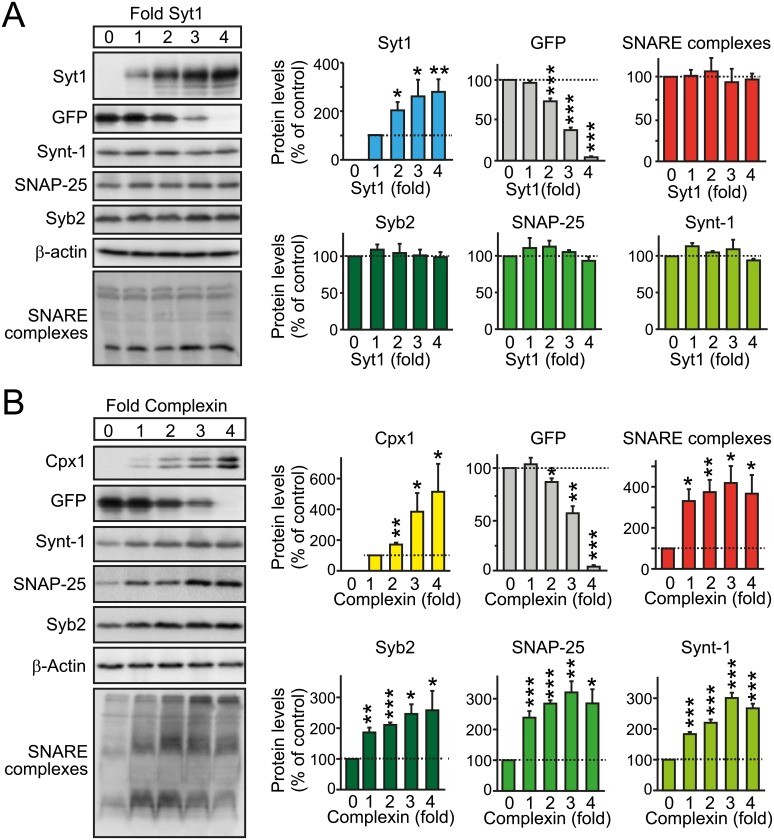
Effect of Syt1 or complexin-1 on SNARE complex assembly when coexpressed in HEK293T cells as a reduced system. **A.** Syt1 alone has no effect on SNARE complex assembly. HEK293T cells were cotransfected with plasmids expressing syntaxin-1 (Synt-1), SNAP-25, and synaptobrevin-2 (Syb-2) in a 1:1:1 ratio, together with increasing amounts of Syt1 expression plasmid (0 to 4-fold), and decreasing amounts of emerald expression plasmid (GFP; 4 to 0-fold), to balance the total amount of transfected DNA. Cell lysates were immunoblotted for Syt1, emerald, SNARE complexes (high molecular mass bands), and total SNARE proteins, followed by quantitation (*n* = 4 independent experiments). **B.** Complexin increases SNARE complex assembly. Same as (A), except that HEK293T cells were co-transfected with an increasing amount of complexin-1 (Cpx1) expression plasmid (*n* = 4 independent experiments). All data are means ± SEM; statistical significance was assessed by Student’s *t* test (* *p* < 0.05, ** *p* < 0.01, *** *p* < 0.001). The data used to make this figure can be found in S1 Data.

How then might Syt1 and Syt7 binding promote RRP maintenance? We first examined whether Syt1 as the paradigmatic synaptotagmin increases SNARE-complex assembly using proteins expressed in cotransfected HEK293 cells but detected no Syt1-dependent change in SNARE complex assembly (Fig 7A). In contrast, complexin strongly promoted SNARE-complex assembly and stabilized individual SNARE proteins (Fig 7B).

**Fig 8:**
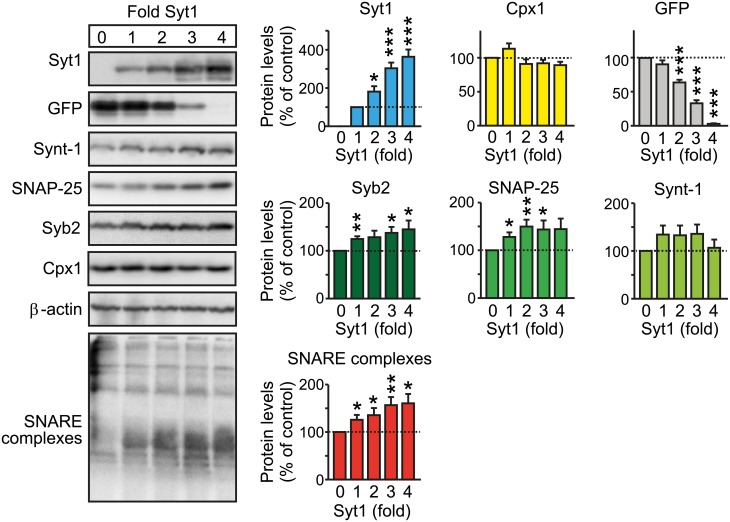
Effect of Syt1 and complexin-1 on SNARE complex assembly when coexpressed in HEK293T cells as a reduced system. Syt1 increases SNARE complex assembly in presence of complexin-1. HEK293T cells were cotransfected with plasmid expressing Synt-1, SNAP-25, Syb-2, and complexin-1 in a 1:1:1:3 ratio, together with an increasing amount of Syt1 expression plasmid (0 to 4-fold), and decreasing amount of emerald expression plasmid (GFP; 4 to 0-fold). Cell lysates were immunoblotted for Syt-1, Cpx1, emerald, SNARE complexes (high molecular mass bands), and total SNARE proteins followed by quantitation (*n* = 6 independent experiments). All data are means ± SEM; statistical significance was assessed by Student’s *t* test (* *p* < 0.05, ** *p* < 0.01, *** *p* < 0.001). The data used to make this figure can be found in S1 Data.

Strikingly, when we expressed increasing amounts of Syt1 in the presence of constant levels of SNARE proteins and complexin, Syt1 significantly increased SNARE-complex assembly above the effect of complexin on SNARE-complex assembly (Fig 8). The effect of Syt1 was independent of Ca^2+^ in the buffer and thus reflects a Ca^2+^-independent binding activity of Syt1 (Fig 8 and S4 Fig). These experiments therefore suggest that Syt1 has a direct Ca^2+^-independent effect on the assembly or stability of SNARE complexes which might account for the function of Syt1 in RRP maintenance, although the atomic mechanisms of this activity by Syt1 are unclear.

### Double Deficiency of Syt1 and Syt7 Does Not Impair the Relative Speed of Vesicle Priming

**Fig 9:**
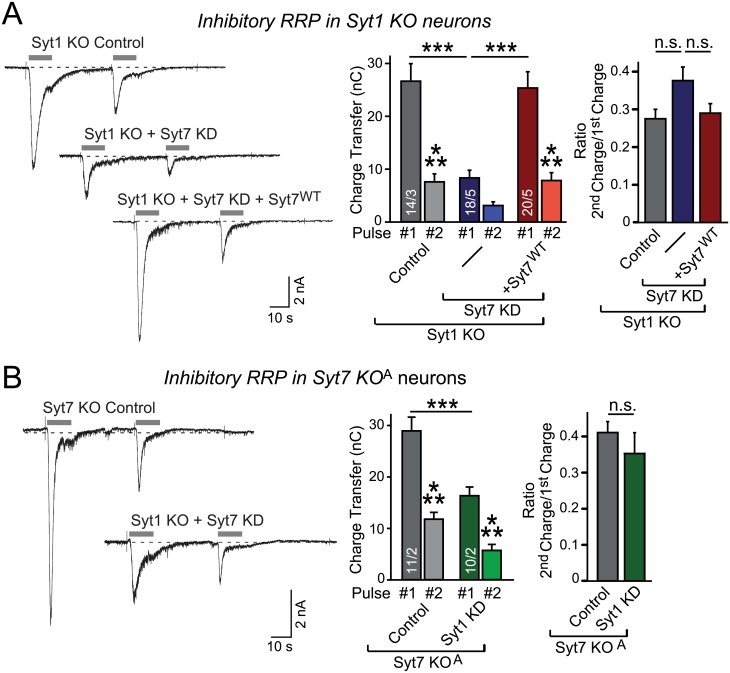
Double deficiency of Syt1 and Syt7 does not impair the priming rate of synaptic vesicles during the recovery of the RRP after depletion. **A & B.** Simultaneous ablation of Syt1 and Syt7 impairs the RRP size but not the relative rate of RRP recovery after RRP depletion at inhibitory synapses. In A, Syt1 KO neurons without or with induction of the Syt7 KD alone or together with the Syt7 rescue were analyzed; in B, Syt7 KO neurons (from KO strain A) without or with Syt1 KD were tested. The RRP was measured during an initial 10 s application of 0.5 M sucrose which also depleted the RRP, and RRP recovery was monitored after 40 s by a second 10 s application of sucrose (gray bars = sucrose applications). Double deficiency of Syt1 and Syt7 resulted in a reduced RRP size with no effect on the relative rate of RRP recovery (left, representative traces; middle, summary graphs of absolute RRP sizes; right, summary graphs of the ratio of 2nd versus 1st RRP to estimate RRP recovery). Recorded currents were not corrected for the significant changes in access resistance similarly observed for all groups (see S4 Fig). All data are means ± SEM; numbers in bars indicate number of neurons/independent cultures analyzed. Statistical significance was assessed by one-way ANOVA (*** *p* < 0.001). The data used to make this figure can be found in S1 Data.

The priming phenotype we observe could be due to a decrease in the capacity of the RRP, or to a deceleration of the rapid refilling of the RRP, a decrease that may manifest as an overall decrease in RRP size as measured by a 30 s application of hypertonic sucrose. Note that the second hypothesis differs from one suggested earlier that the Syt7 KO causes a decrease in the speed of synaptic vesicle priming [65]—we have not been able to observe any effect of the Syt7 KO alone on priming in either the current experiments (Fig 1) or previous studies [28,29]. However, the lack of an effect of the single Syt7 KO on priming does not rule out a possible effect of the double Syt1/7 deficiency on the priming rate, which we tested by the standard approach of applying two short pulses (10 s) of hypertonic sucrose separated by 40 s [66–68]. We again found that the Syt1/7 double deficiency had no effect on the rate of RRP replenishment (Fig 9). The initial RRP size was greatly decreased by the Syt1/7 double deficiency consistent with our previous results (Fig 1); despite its smaller size, the RRP did not refill faster in Syt1/7 double-deficient synapses than in WT synapses. Note, however, that due to the stress of the double pulse of hypertonic sucrose, the access resistance is increased significantly during the second sucrose pulse (S5 Fig), which suggests that these experiments, although standard in the field, should be considered with caution and can only provide clues to the relative recovery rate of the RRP after depletion.

## Discussion

**Fig 10:**
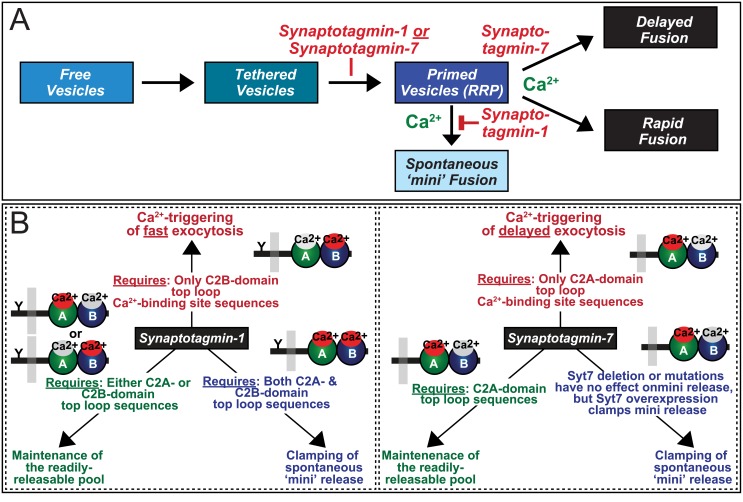
Summary of structure–function relations of Syt1 and Syt7 in Ca^2+^ triggering of exocytosis, priming, and clamping of spontaneous mini release. **A.** Schematic diagram of the different stages of Ca^2+^-triggered synaptic vesicle exocytosis and of the functions of Syt1 and Syt7 in the maintenance of the RRP size, clamping spontaneous mini release (which is in itself Ca^2+^-dependent via the actions of an as yet unidentified Ca^2+^ sensor [49]), and Ca^2+^ triggering of delayed and fast release. **B.** The three different functions of Syt1 (top) and Syt7 (bottom) are depicted by arrows; the C2 domain requirement for each function is described on top of the arrows. In addition, schematic diagrams of the Syt1 and Syt7 domain structures are shown, with the top-loop Ca^2+^-binding sequences of a given C2 domain that is required for a particular function indicated in red.

Synaptotagmins are well-characterized Ca^2+^ sensors for exocytosis not only in neurons where Ca^2+^ triggers neurotransmitter release by binding to synaptotagmins, but also in other cell types with Ca^2+^-triggered exocytosis, such as endocrine cells and mast cells [69–71]. In neurons, three synaptotagmins (Syt1, Syt2, and Syt9) mediate fast synchronous neurotransmitter release, while Syt7 functions in delayed asynchronous release [3]. Moreover, the same synaptotagmins also act as Ca^2+^ sensors for neuropeptide exocytosis in the same manner, and Syt10—which differs structurally from Syt1, Syt2, Syt7, and Syt9—mediates Ca^2+^-dependent IGF-1 exocytosis [44,72]. In addition to their role as Ca^2+^ sensors for exocytosis, synaptotagmins are clamps of exocytosis that suppress spontaneous “mini” neurotransmitter release from presynaptic terminals; as a result of the loss of this function, deletion of Syt1 or Syt2 causes an increase in mini release [26,39,48]. Unexpectedly, we here find that Syt1 and Syt7 perform a third, independent function in neurotransmitter release: enabling a normal RRP size (summarized in Fig 10); as a result of the loss of this function, deletion of both Syt1 and Syt7 causes a decrease in RRP size (Figs 1–3). Thus, the current findings extend the functions of synaptotagmins to steps upstream of Ca^2+^ triggering of release and suggest that synaptotagmins, their simple domain structure notwithstanding, perform three sequential roles in neurotransmitter release similar to the even smaller complexins that act as obligatory cofactors of synaptotagmins in all of these functions [3].

### The RRP Function of Syt1 and Syt7

The conclusion that Syt1 and Syt7 are required for maintaining a normal RRP size is based on the observation that simultaneous loss-of-function of both Syt1 and Syt7 decreased the RRP size, whereas loss-of-function of only Syt1 or Syt7 alone had no effect on RRP size (Figs 1–3). The fact that the RRP phenotype—different from the Ca^2+^-triggering phenotype [22,28]—was only observed upon ablating both Syt1 and Syt7, and that it was rescued by either WT Syt1 or Syt7 but not by mutant Syt1 or Syt7 with substitutions in their top-loop sequences, demonstrates the specificity of the phenotype. We measured the RRP size via the release elicited by a brief application of hypertonic sucrose [12]. A phenotype observed with this procedure could also have been due to changes in synapse number or synapse structure, but we detected no alterations in either parameter as assessed by immunocytochemistry and EM (Fig 5) [28]. We also confirmed that under our conditions, sucrose-induced synaptic responses reflect a Ca^2+^-independent priming process, showing that the involvement of Syt1 and Syt7 in priming cannot operate by low-level Ca^2+^ binding to Syt1 or Syt7 (S1 Fig).

Our results raise the important question whether the priming and Ca^2+^-triggering functions of Syt1 and Syt7 are distinct or identical, i.e., do Syt1 and Syt7 really function as Ca^2+^ sensors at all, or only as priming factors? This question is prompted by our surprising observation that mutations in the top-loop sequences of Syt1 and Syt7, which contain their Ca^2+^ binding sites block their priming functions, raising the possibility that what was previously observed as a Ca^2+^-triggering function may in fact represent a priming function. However, three observations document that synaptotagmins perform distinct functions in RRP maintenance and Ca^2+^ triggering. First, the Ca^2+^-triggering functions of Syt1 and Syt7 are clearly apparent in their individual KOs [22,27–29,39], whereas their priming functions are not. The Ca^2+^-triggering phenotype of the Syt1 KO or KD includes a block of nearly all fast synchronous release, while the Ca^2+^-triggering phenotype of the Syt7 KO or KD impairs delayed asynchronous release [27–29]. Second, Syt1 requires distinct sequences for its priming and Ca^2+^-triggering functions—the former can be mediated by either the C2A or the C2B domain top-loop sequences, whereas the latter is blocked selectively by mutations in the C2B-domain top-loop sequences (Fig 5) [60–62]. Thus, Syt1 with a C2B domain mutation can still mediate priming but not Ca^2+^ triggering, demonstrating that the priming and Ca^2+^-triggering functions of Syt1 are mechanistically distinct. Third, changing the Ca^2+^-binding affinity of Syt1 changes the Ca^2+^ dependence of fast neurotransmitter release in a parallel fashion [23,54]. Together, these points establish that priming and Ca^2+^ triggering are separate processes for synaptotagmins.

What exactly do synaptotagmins do with respect to the RRP? The phenotype we observe consists of a major decrease in the RRP size without a change in the rate of RRP replenishment, suggesting that the capacity of the RRP is decreased. Vesicles in the RRP are thought to be docked by partial or complete assembly of SNARE complexes [4]. Syt1 is known to bind to assembled SNARE complexes in both a Ca^2+^-dependent and a Ca^2+^-independent manner [54], and we show here that Syt7 also binds to SNARE complexes (Fig 6), and that Syt1 can promote SNARE complex assembly in the presence of complexin (Figs 7 and 8). It is therefore tempting to speculate that synaptotagmins bind to and even promote the formation of assembled SNARE complexes of vesicles in the RRP in a Ca^2+^-independent manner, and that the RRP size decreases in the absence of synaptotagmins because their binding to assembling SNARE complexes stabilizes the primed state of synaptic vesicles. This model agrees well with recent crystallography studies [64], although the precise atomic configuration of the synaptotagmin complexes with SNARE complexes remains unclear, and it seems likely that there are multiple binding interfaces that may also serve to multimerize assemblies of synaptotagmins with SNARE–complexin complexes. Thus, we propose that other proteins—in particular Munc13 [73]—catalyze the actual priming reaction by facilitating SNARE complex assembly, but that synaptotagmins stabilize primed vesicles in the RRP, thereby enabling a normal RRP size.

### Relation to Previous Functional Studies

The Syt7 KO was reported to decrease the speed of vesicle priming [65], but this change was not detected in other studies [29], including the present work (Fig 9). Moreover, it is hard to imagine how Syt7, which is highly homologous to other synaptotagmins, could have a completely different function from other synaptotagmins in presynaptic terminals, but a similar function in neuroendocrine cells [30,35]. Overexpression of Syt2 fragments in the calyx of Held synapse decreased neurotransmitter release, which was interpreted as a change in vesicle positioning and a disruption of the RRP [74], but it is difficult to assess the molecular consequences of introducing large amounts of Syt2 fragments into a nerve terminal where such fragments could, for example, interfere with release by indiscriminately coating lipid membranes, or by binding to all assembling SNARE complexes. Thus, the mechanism of the inhibition of release by the overexpressed Syt2 fragments is difficult to interpret vis-à-vis priming. Finally, mutations in SNAP-25 that affect Syt1 interactions were shown in chromaffin cells to decrease priming of neuroendocrine vesicles [75]. Although this observation is consistent with a role of synaptotagmins in priming, it does not exclude the possibility that other actions by SNAP-25—which is a central component of SNARE complexes—are also affected by the SNAP-25 mutations, and it does not directly address the question whether synaptotagmins function in maintaining the RRP size. Thus, our data are broadly consistent with previous studies.

### The Triple Life of Synaptotagmins

Viewed together with earlier studies, our results suggest that synaptotagmins mediate three distinct sequential processes in presynaptic terminals: maintenance of the normal capacity of the RRP, clamping of spontaneous mini release, and Ca^2+^ triggering of evoked release (Fig 10). Syt1 likely functions in these three processes by separate molecular mechanisms, because the function of Syt1 in these three processes exhibits distinct C2 domain requirements (Fig 5) [28,62], and it seems likely that this also applies for Syt7. Although Syt1 and Syt7 perform similar overall functions, they exhibit differences not only in their C2 domain requirements but also in their relative importance for Ca^2+^ triggering and clamping. In most synapses, the Ca^2+^-triggering phenotype of the Syt7 KO or KD is small, whereas that of the Syt1 KO or KD is robust. Moreover, the Syt7 KO or KD has no effect on mini release, whereas the Syt1 KO or KD unclamps most synapses. Overall, however, these differences appear minor compared to the functional overlap between Syt1 and Syt7, as documented by their complete redundancy in RRP maintenance (Figs 1–3), and the rescue of the clamping phenotype in Syt1 KO synapses by Syt7 overexpression [28].

We propose that Syt1 and Syt7 act sequentially in multiple processes by overlapping mechanisms. Initially, synaptic vesicles are tethered to the active zone by the binding of the active zone protein RIM to the synaptic vesicle GTP-binding protein Rab3 or Rab27 [8,9]. At the same time, syntaxin-1 containing bound Munc18 is converted from a “closed” to an “open” conformation, most likely by RIM-bound Munc13 [73]. Together, these two processes enable SNARE complex assembly from vesicular synaptobrevin with plasma membrane syntaxin-1 and SNAP-25, whereupon complexin and synaptotagmins bind to the partially assembled SNARE complexes in a Ca^2+^-independent manner. The binding of complexin and synaptotagmins to assembling SNARE complexes is proposed to both boost and stabilize SNARE complex assembly as evidenced by the effect of complexin and Syt1 on SNARE complex assembly in transfected HEK293 cells as a reduced system (Figs 7 and 8) and to mediate clamping of mini release. Subsequent Ca^2+^ influx into the nerve terminal during an action potential is mediated by Ca^2+^ channels that are recruited into a location next to docked vesicles by RIMs [9]. The highly localized rapid rise in Ca^2+^ leads to Ca^2+^ binding to Syt1 and Syt7, which (i) causes a rearrangement of synaptotagmin-binding to SNAREs, such that the central α-helix of complexin is displaced from the SNARE complex [41] and (ii) induces synaptotagmin binding to phospholipids that may cause a mechanical “pull” on these membranes [3,76,77]. Together, these two Ca^2+^-triggered molecular events may enable completion of SNARE complex assembly, thereby opening the fusion pore with some contribution from Munc18.

This model accounts for all of the observations described here and additionally agrees with the finding that complexin, the partner for Syt1 in synapses, is also required for vesicle priming, clamping, and Ca^2+^ triggering [41,42,45–47,78–81]. However, this model does not explain the differential C2 domain requirements and distinct kinetics of the Ca^2+^-triggering functions of Syt1 and Syt7. Although this model is consistent with the notion that a macromolecular assembly composed of a synaptotagmin/complexin/SNARE complex serves as a molecular platform for all three functions of synaptotagmins, the precise atomic structure of this assembly has remained elusive. Two recent biophysical studies describe structures of the complex of the C2AB domain fragment of Syt1 with SNARE complexes but come to different conclusions about the Syt1 sequences that mediate this complex [63,64]. Moreover, a recent crystal structure reveals multiple interfaces with large contact areas [64], suggesting that synaptotagmins may engage in multiple different complexes with SNARE complexes and possibly thereby multimerize SNARE complexes. Furthermore, none of the biophysical models involves the top Ca^2+^-binding loops of Syt1 that were functionally implicated in the present study. Finally, mutations in residues involved in the various interfaces all interfere with Ca^2+^-triggered release, suggesting that all of these interfaces are important. In view of these results it is, at present, not yet possible to suggest a precise atomic model for synaptotagmin function in concert with SNARE complexes and complexin, but it seems likely that multiple complexes involving all three components—synaptotagmins, complexins, and SNAREs—execute the three functions of synaptotagmins that enable synaptotagmins to act as the central regulators of exocytosis.

## Methods

### Ethics

This study was performed in strict accordance with the recommendations in the Guide for the Care and Use of Laboratory Animals of the National Institutes of Health. The protocol was approved by the committee on the Ethics of Animal Experiments (IACUC) of the Stanford University (Protocol Numbers 29589 and 18846).

### Mouse Husbandry, Reagents, and Plasmid Constructs

All mouse lines used here were reported previously [28]. Mice were bred using standard procedures and are available at Jackson Labs. All plasmids, including the Syt1 and Syt7 KD lentiviral vectors, and antibodies used were also described previously [28,57]. For the KD experiments, we used the following oligonucleotide sequences: Syt7, KD606 AAAGACAAGCGGGTAGAGAAA and KD607 GATCTACCTGTCCTG GAAGAG; Syt1, GAGCAAATCCAGAAAGTG CAA.

Hippocampal neurons were cultured from WT, Syt1 KO, and Syt7 KO mice as described [39]. Briefly, hippocampi were dissected from newborn pups, dissociated by papain digestion, and plated on Matrigel-coated glass coverslips. Neurons were cultured for 14–16 days in vitro in MEM (Gibco) supplemented with B27 (Gibco), glucose, transferrin, fetal bovine serum, and Ara-C (Sigma).

### Lentivirus Production and Infection of Cultured Neurons

The production of lentiviruses and infection of neurons with lentiviruses have been described [41]. Briefly, supernatant with viruses was collected 48 hr after cotransfection of human embryonic kidney 293T cells with the lentiviral vector and three packaging plasmids. This supernatant was used to infect hippocampal neuronal cultures at DIV4, and cultures were used for biochemical or physiological analyses at DIV14–16.

Electrophysiological recordings in cultured neurons were performed essentially as described [41,82]. Briefly, the resistance of pipettes filled with intracellular solution varied between 2–3 MOhm and the series resistance was 7–10 MOhm. Synaptic currents were monitored with a Multiclamp 700B amplifier (Molecular Devices). The frequency, duration, and magnitude of the extracellular stimulus were controlled with a Model 2100 Isolated Pulse Stimulator (A-M Systems, Inc.) synchronized with the Clampex 9 or 10 data acquisition software (Molecular Devices). Evoked synaptic responses were triggered by a bipolar electrode. The whole-cell pipette solution contained (in mM) 135 CsCl, 10 HEPES, 1 EGTA, 1 Na-GTP, 4 Mg-ATP, and 10 QX-314 (pH 7.4, adjusted with CsOH). The bath solution contained (in mM) 140 NaCl, 5 KCl, 2 MgCl_2_,10 HEPES, 10 glucose (pH 7.4, adjusted with NaOH) and various concentrations of free extracellular Ca^2+^ (2 mM except otherwise stated). AMPAR-mediated EPSCs were isolated pharmacologically with picrotoxin (50 μM) and AP-5 (50 μM), and recorded at a −70 mV holding potential, NMDAR-mediated EPSCs with picrotoxin (50 μM) and CNQX (20 μM) and recorded at a +40 mV holding potential, and IPSCs with CNQX (20 μM) and AP-5 (50 μM) and recorded at a −70 mV holding potential; all drugs were applied to the bath solution. Note that IPSCs were recorded with a high internal Cl^-^ solution, resulting in large inward currents. mIPSCs and mEPSCs were monitored in the presence of tetrodotoxin (1 μM) in addition to the compounds listed above. Miniature events were analyzed in Clampfit 9.02 (Molecular Devices) using the template matching search and a minimal threshold of 5 pA and each event was visually inspected for inclusion or rejection. Because the standard software was unable to measure the vastly increased mIPSC frequency in Sy1 KO neurons, we wrote a custom Matlab algorithm that analyzed the first derivative of the trace to identify potential events, followed by screening for the rise time and amplitude. For Ca^2+^ titrations, eIPSCs were measured for each cell at multiple Ca^2+^ concentrations starting at 2 mM Ca^2+^, followed by measurement of the higher then lower Ca^2+^ concentration points. Sucrose release was triggered by a 30 s application of 0.5 M sucrose and was measured in the presence of 1 μM tetrodotoxin plus additional inhibitors; the synaptic charge transfer was integrated over 30 s. For all electrophysiological experiments, the experimenter was blind to the condition or genotype of the cultures analyzed.

### Immunoprecipitation and Quantitative Immunoblotting

Cultured neurons were solubilized in PBS (with 1 mM CaCl_2_, 0.2% Triton X-100, pH 7.4) supplemented with protease inhibitors (Roche) for 1 h. The lysate was cleared by centrifugation at 16,000 g for 10 min at 4°C. Immunoprecipitations were performed by incubating with polyclonal antibodies to syntaxin-1 (438B) or preimmune sera for 1 h at 4°C, followed by incubation with 15 μl of a 50% slurry of protein-A Sepharose beads (GE Healthcare) for 2 h at 4°C. Beads were washed 4x with 1 ml extraction buffer, bound proteins were eluted with 2× SDS sample buffer containing 100 mm DTT and boiled for 20 min at 100°C. Coprecipitated proteins were separated by SDS-PAGE followed by detection with monoclonal antibodies against an HA epitope included in Syt1 (HA.11; 16B12, Covance) and synaptobrevin-2 (cl. 69.1, Synaptic Systems). To allow for quantitative detection, dye-conjugated secondary antibodies were used (IRDye 800CW Donkey anti-Mouse IgG, Li-cor), membranes were scanned in an Odyssey scanner (Li-cor), and quantification was performed using Image Studio software (Li-cor).

### SNARE complex Assembly Studies in Transfected HEK293T Cells

For complexin titration experiments, HEK293T cells were cotransfected with pCMV5 syntaxin-1a, pCMV5 SNAP-25a, pCMV5 synaptobrevin-2 (1:1:1), and an increasing amount of pCMV5 complexin-1 (0 to 4-fold). Total DNA was kept constant by balancing the complexin-1 plasmid with pCMV5 emerald (4 to 0-fold). For Syt1 titration experiments, HEK293T cells were cotransfected as above, except that pCMV5 complexin-1 was replaced with pCMV5 Syt1. For Syt1 titration experiments in presence of complexin-1, HEK293T cells were cotransfected with pCMV5 syntaxin-1a, pCMV5 SNAP-25a, pCMV5 synaptobrevin-2, pCMV5 complexin-1 (1:1:1:3), and an increasing amount of pCMV5 Syt1 (0 to 4-fold). Total DNA was kept constant by balancing the Syt1 plasmid with pCMV5 emerald (4 to 0-fold). Two days after transfection, cells were harvested by solubilization for 1h at 4°C in 0.1% Triton X-100 in PBS, supplemented with protease inhibitors (Roche). For Syt1 titration experiments in presence of complexin-1, cells were solubilized in 0.1% Triton X-100 in TBS with or without addition of 2 mM CaCl_2_, supplemented with protease inhibitors (Roche). Cell lysates were analyzed either directly by quantitative immunoblotting (for SNARE complexes; high molecular mass bands), or were first boiled for 20 min at 100°C (for total SNARE proteins, complexin-1, Syt1, and emerald).

Antibodies used: synaptobrevin-2 (cl. 69.1, SySy), SNAP-25 (cl. 71.1, SySy; SMI81, Sternberger Monoclonals), syntaxin-1 (HPC-1, SySy), complexin-1 (122002, SySy), Syt1 (V216), GFP (T3743), β-actin (A1978, Sigma).

### EM

Cultured neurons were fixed with prewarmed 2% glutaraldehyde in 0.1 M sodium cacodylate buffer, pH 7.4 at room temperature for 1 h, then stored in 0.2% glutaraldehyde in cacodylate buffer. Samples were postfixed in 0.5% OsO_4_, 0.8% potassium ferricyanide (K_3_FeCN_6_) in the same buffer at room temperature for 30 min. Specimens were stained en bloc with 2% aqueous uranyl acetate for 15 min, dehydrated in a graded series of ethanol to 100% and embedded in Poly/bed 812 for 24 hr. Thin sections (60 nm) were poststained with uranyl acetate and lead citrate. Samples were examined with a FEI Tecnai transmission electron microscope at 80 kV accelerating voltage; digital images were captured with an Olympus Morada CCD camera. Quantitative analyses were conducted on digital electron micrographs with magnification of 30,000x, and the following sample size (images/synapses analyzed): Control, 59/83; Syt7 KD 77/109; Syt7 KD+WT rescue 49/71; Syt7 KD+5DA rescue 28/49. The number of docked vesicles (defined as vesicles touching the presynaptic plasma membrane), vesicles at the active zone (defined as vesicles within 100 nm distance from the presynaptic membrane), number of vesicles per bouton, PSD length and bouton area were analyzed in ImageJ. Both “docked vesicles” and “vesicles at the active zone” were analyzed to sensitively detect any possible docking phenotype. Images were analyzed by an experimenter blind to the condition and were repeated two times in independent batches of cultures; the results observed were comparable.

### Statistics

All data are shown as means ± SEM, and all statistical analyses were performed by one-way ANOVA or Student’s *t* test.

## Supporting Information

S1 Data Excel file containing data and statistics used in all figures. (XLSX)

S1 Fig RRP replenishment by vesicle priming at inhibitory hippocampal synapses is Ca^2+^-independent. **A.** Cultured neurons were incubated in a Ca^2+^-free buffer supplemented with 10 mM BAPTA-AM for 45 min. RRP release was triggered with hypertonic sucrose at the two times indicated by the gray bars. Expanded trace at the bottom left shows that under the conditions of the experiment with low extracellular and chelated intracellular Ca^2+^, spontaneous mini release is almost completely suppressed. A representative cell from multiple recordings is shown. **B.** Comparison of the size of the RRP and the relative rate of RRP recovery after depletion between synapses at physiological Ca^2+^ concentrations and synapses in which all intracellular Ca^2+^ is chelated with BAPTA as described for the representative experiment in panel A. The RRP is depleted by an initial 10 s application of hypertonic sucrose, and recovery is monitored after 60 s by application of a second 10 s pulse of hypertonic sucrose. All data are means ± SEM; numbers in bars indicate number of neurons or independent cultures analyzed. The data used to make this figure can be found in S1 Data.
(EPS)

S2 Fig A naturally occurring Syt7 splice variant containing a longer linker region fully rescues evoked release and synaptic vesicle priming in Syt1/7 double-deficient neurons, whereas a truncation mutant of Syt7 lacking the C2B domain does not rescue. **A & B.** Hippocampal neurons cultured from Syt1 KO mice were infected at DIV4 with control or Syt7 KD lentiviruses coexpressing the long-linker Syt7 (Syt7^L^) or a truncated Syt7 that lacks the C2B-domain (Syt7^C2A^). At DIV14–16, evoked IPSCs were measured during a 10 Hz, 1 s stimulus train (tick marks; A), and the size of the RRP was estimated by stimulating release using an application of 0.5 M sucrose for 10 s (gray bars in representative traces on the left; B). IPSCs were monitored in the presence of CNQX (20 μM) and AP5 (50 μM); in addition, RRP measurements were performed with tetrodotoxin (1 μM). All data are means ± SEM; numbers in bars indicate number of neurons or independent cultures analyzed. Statistical significance was assessed by one-way ANOVA (* *p* < 0.05, *** *p* < 0.001). The data used to make this figure can be found in S1 Data.
(EPS)

S3 Fig Swapping the critical Syt1 and Syt7 top-loop sequences that contain their Ca^2+^ binding sites does not dramatically alter Syt1 and Syt7 function, whereas a point mutation adjacent to the C2A-domain Ca^2+^-binding sequence (R228Q) greatly impairs Syt7 function. **A.** Diagram showing the alignment of the C2AB domain regions of Syt1 and Syt7. The blue bars show the top-loops of the C2 domains; each C2 domain containing three top-loops, which mediate Ca^2+^ binding. The location of the D227 and R228 residues in Syt7 is shown in bold, while the mutant versions of these amino acids (N and Q) are shown in purple. For the swapped loops constructs, the mutation introduced in each loop are indicated on top. Thus, the Syt1^Loop7^construct is identical to Syt1 except for the top blue residues indicated to convert the loops to the Syt7 sequence. For the Syt7^Loop1^construct, the mutations introduced are shown below the alignment by the red residues. **B & C.** The R228Q mutation in Syt7, equivalent to the R233Q mutation in Syt1, severely affects Syt7 function in both evoked release (B) and RRP maintenance (C). The D227N mutation, equivalent to the D232N mutation in Syt1, or the loops swap mutants as in (A), supports evoked release (B) and RRP (C) similarly to WT, Syt7, or Syt1. Hippocampal neurons were cultured from Syt1 KO mice and infected with control lentivirus, or Syt7 KD lentiviruses coexpressing the indicated Syt1 and Syt7 mutants. Evoked IPSCs triggered by a 10 Hz, 1 s stimulus train (tick marks) were monitored in CNQX (20 M) and AP5 (50 M) (B). To measure the RRP (C), a brief pulse of 0.5 M sucrose was delivered and IPSCs were measured in the presence of TTX (1 M), CNQX, and AP5. Representative traces are shown on the left and summary graphs on the right. All data are means ± SEM; numbers in bars indicate number of neurons or independent cultures analyzed. Statistical significance was assessed by one-way ANOVA (* *p* < 0.05, ** *p* < 0.01, *** *p* < 0.001). The data used to make this figure can be found in S1 Data.
(EPS)

S4 Fig Ca^2+^ does not enhance the effect of Syt1 on SNARE complex assembly in transfected HEK293T cells. **A & B.** HEK293T cells were cotransfected with plasmid-expressing syntaxin-1a (Synt-1), SNAP-25, synaptobrevin-2 (Syb-2), and complexin-1 (Cpx1) in a constant 1:1:1:3 ratio, together with an increasing amount of Syt1 expression plasmid (0 to 4-fold) and a decreasing amount of emerald expression plasmid (GFP; 4 to 0-fold) to balance the total DNA amount. Cells were lysed in 0.1% Triton X-100 containing 2 mM CaCl_2_, and lysates were immunoblotted for Syt1, Cpx1, emerald, SNARE complexes, and total SNARE proteins (A), followed by quantitation using phosphoimager detection (B). SNARE complexes were quantified as the multiple bands of SDS-resistance complexes on the gel. Data are means ± SEM (* *p* < 0.05, ** *p* < 0.01, *** *p* < 0.001, by Student’s *t* test; *n* = 6 independent experiments). The data used to make this figure can be found in S1 Data.
(EPS)

S5 Fig The double sucrose application used for measuring the priming rate of synaptic vesicles increases the access resistance approximately 2-fold in control and Syt1/7 double-deficient neurons. **A & B.** Measurements of the access resistance in Syt1 KO neurons without or with Syt7 KD alone or Syt7 KD together with Syt7 rescue (A) and in Syt7 KO neurons (from KO strain A) without or with the Syt1 KD (B). Access resistance was measured with a 5 mV hyperpolarizing step 100 ms before each sucrose pulse (see Fig 6; left summary graphs show the absolute access resistance values, while right summary graphs show the ratio of access resistances). All data are means ± SEM; numbers in bars indicate number of neurons or independent cultures analyzed. Statistical significance was assessed by one-way ANOVA (* *p* < 0.05, *** *p* < 0.001). The data used to make this figure can be found in S1 Data.
(EPS)

## Abbreviations

EM: electron microscopy
EPSC: excitatory postsynaptic current
IPSC: inhibitory postsynaptic current
KD: knockdown
KO: knockout
mEPSC: miniature excitatory postsynaptic current
mIPSC: miniature inhibitory postsynaptic current
NMR: nuclear magnetic resonance
PSD: postsynaptic density
RIMs: Rab3-interacting molecules
RRP: readily-releasable pool
SEM: standard error of the mean
SM: Sec1/Munc18-like
SNARE: soluble NSF-attachment protein receptor
Syt1: synaptotagmin-1
Syt7: synaptotagmin-7
WT: wild-type
